# The function of Foxp1 represses β-adrenergic receptor transcription in the occurrence and development of bladder cancer through STAT3 activity

**DOI:** 10.1515/med-2023-0647

**Published:** 2023-08-24

**Authors:** Zhenshan Ding, Binbin Jiao, Xuelong Chen, Xing Chen, Yangtian Jiao, Jianfeng Wang, Xiaofeng Zhou

**Affiliations:** Department of Urology, China-Japan Friendship Hospital, No. 2, Yinghua East Road, Chaoyang District, Beijing 100029, China; Department of Clinical Medicine, Peking University China-Japan Friendship School, Beijing 100029, China; Department of Urology, China-Japan Friendship Hospital, Beijing 100029, China

**Keywords:** Foxp1, β-AR, STAT3, bladder cancer, Warburg effect

## Abstract

Bladder cancer is a common malignant tumor. FOXP1 has been found to be abnormally expressed in tumors such as renal cell carcinoma and endometrial cancer. Here, this investigated the biological roles of Foxp1 in the occurrence and development of bladder cancer. Patients with bladder cancer were obtained from China-Japan Friendship Hospital. Bladder cancer cell lines (5637, UMUC3, J82, and T24 cell) were used in this experiment. Foxp1 mRNA and protein expression levels in patients with bladder cancer were increased, compared with paracancerous tissue (normal). OS and DFS of Foxp1 low expression in patients with bladder cancer were higher than those of Foxp1 high expression. Foxp1 promoted bladder cancer cell growth *in vitro* model. Foxp1 increased the Warburg effect of bladder cancer. Foxp1 suppressed β-adrenoceptor (β-AR) expression *in vitro* model. ChIP-seq showed that Foxp1 binding site (E1, TTATTTAT) was detected at −2,251 bp upstream of the β-AR promoter. β-AR Reduced the effects of Foxp1 on cell growth *in vitro* model. β-AR reduced the effects of Foxp1 on the Warburg effect *in vitro* model by STAT3 activity. Taken together, our findings reveal that Foxp1 promoted the occurrence and development of bladder cancer through the Warburg effect by the activation of STAT3 activity and repressing β-AR transcription, and which might serve as an important clue for its targeting and treatment of bladder cancer.

## 1 Introduction

Bladder cancer is one of the most common malignant tumors in the urogenital system, which ranks ninth among the most common malignant tumors in Europe and the United States [1]. In recent years, the incidence and mortality of bladder cancer in China are on the rise in China [2]. Bladder cancer can occur at any age, with a high incidence in men over 65 years of age [3]. In addition, the incidence ratio of males to females is 3:1–4:1 [4]. Clinically, about 70% of bladder cancers are non-invasive and 30% are invasive [5,6]. Among them, non-muscular invasive bladder cancer is characterized by a high recurrence rate and low mortality, whereas approximately 50% of invasive bladder cancer is potentially fatal [5].

Because of the uncertainty of the immune system in regulating the growth of solid tumors, whether the stress hormones released by the sympathetic nervous system can directly affect the proliferation and metastasis potential of tumor cells has become a research hotspot in recent years. A large number of studies have found that the increased neuropeptides and neurotransmitters produced by the body under stress can change the biological behavior of tumor cells in various stages of tumor progression and metastasis. The neurotransmitter released by the sympathetic postganglionic fibers of the human body is mainly norepinephrine, which acts on β-adrenoceptor (β-AR) and plays a regulatory role. Research shows that the β-AR signal pathway is closely related to the occurrence and development of tumors.

β-AR is a class of intracellular proteins that plays a role in the invasion and metastasis of tumor cells by mediating the desensitization of seven transmembrane-coupled receptors [7]. It can also regulate the corresponding signal pathways through the phosphorylation, ubiquitination, or intracellular localization of other signal molecules [8]. Moreover, β-AR regulates the life course of tumor cells by playing different roles in the corresponding signal pathways [9].

FOXP1, a member of the FOX family, is involved in cardiomyocyte development, immune B cell differentiation, and motor neuron diversity [10]. It is found that FOXP1 is abnormally expressed in various tumors such as breast cancer, lung cancer, bladder cancer, and lymphoma, which may become a tumor marker for clinical practice [11,12]. PBRM1 is a chromatin complex protein that is abnormally expressed in tumors such as renal cell carcinoma and endometrial cancer, which is associated with the occurrence and development of tumors [13,14]. Here, we investigate the biological roles of Foxp1 in the occurrence and development of bladder cancer.

## 2 Materials and methods

### 2.1 Experimental clinical medicine

Patients with bladder cancer (*n* = 41) and paracancerous tissue (*n* = 41) were obtained from the China-Japan Friendship Hospital from May 2017 to Mar 2018. Tissue samples were collected and saved at −80°C. Overall survival and disease-free survival were executed and followed for 3 years.


**Ethics approval and consent to participate:** All patients were informed and signed informed consent voluntarily. This study was approved by the ethics committee of the China-Japan Friendship hospitals and complied with the guidelines outlined in the Declaration of Helsinki were followed. The written consent was received from all participants.

### 2.2 Cell culture and transfection

Normal bladder epithelial cells (GES-1 cell), and bladder cancer cell lines (5637, UMUC3, J82, and T24 cell) were cultured in RPMI 1640 medium (Gibco, Carlsbad, CA, USA) supplemented with 10% fetal calf serum (FCS, Gibco, Carlsbad, CA, USA) in a humidified atmosphere of 5% CO_2_ at 37°C. Plasmids were transfected into cell using Lipofectamine 2000 as literature [15].

### 2.3 Microarray analysis

Total RNA was extracted from serum samples, and the amount of RNA was quantified by use of NanoDrop 1000. The total RNA of each sample was used for reverse transcription using an Invitrogen SuperScript double-stranded cDNA synthesis kit. Double-stranded cDNA was executed with a NimbleGen one-color DNA labeling kit and then executed for array hybridization using the NimbleGen hybridization system and washing with the NimbleGen wash buffer kit. Axon GenePix 4000B microarray scanner (Molecular Devices) was used for scanning as literature [16].

### 2.4 Real-time quantitative reverse transcription-polymerase chain reaction (RT-PCR)

Total cellular RNA was extracted using Trizol (Thermo Fisher Scientific) from serum, lung tissue, or cells. RNA was then reverse transcribed into cDNA using Moloney murine leukemia virus reverse transcriptase. The mRNA expression was quantified using 7500 Fast Real-Time PCR System (Applied Biosystems, Foster City, CA) and SYBR® Premix Ex TaqTM II (Takara, Japan). Relative gene expression was normalized to GAPDH.

### 2.5 Cell viability assay

After 48 h of transfection, 1 × 10^3^ cells/well were seeded in a 96-well plate. After culturing at the indicated time (0, 6, 12, 24, and 48 h), the cellular proliferation was detected using Cell Counting Kit-8 (CCK-8, Beyotime, Beijing, China) by CellTiter-GloR Luminescent Cell Viability Assay according to manufacturer’s

### 2.6 EdU assay

About 50 μM of EdU was used to incubate at 37°C for 1 h, fixed with 4% paraformaldehyde for 15 min, and then stained with DAPI for 15 min. The images were captured under fluorescence microscopy (Nikon, Melville, NY, US).

### 2.7 Western blot analysis

Total protein lysates from cell samples were solubilized in SDS-PAGE sample buffer using Radio‐Immunoprecipitation Assay and PMSF reagent, separated on a 10% SDS-polyacrylamide gel, and transferred electrophoretically onto polyvinylidenedifluoride (PVDF) membranes. The membranes were blocked with non-fat-milk (5%) for 2 h at room temperature and incubated with anti-Foxp1, anti-β-AR, anti-STAT3, anti-p-STAT3, and anti-β-actin antibodies at 4°C. The membranes were incubated with horseradish peroxide-coupled sheep anti-mouse secondary antibody at room temperature for 2 h. The bound antibodies were detected using enhanced chemiluminescence (ECL) with β-actin used as a control. Band intensities were quantified using Image J software.

### 2.8 ChIP-seq analysis assay

Cells (1 × 10^4^) were cross-linked with 1% formaldehyde at room temperature for 10 min and incubated with 125 mM glycine for 5 min. Cells were sonicated on ice to generate DNA fragments. The fragmented chromatin fragments were immunoprecipitated with protein A/G magnetic beads (Millipore) coupled with anti-Foxp1 antibody at 4°C overnight with rotation. Input DNA libraries were generated using the NEBNext Ultra II DNA Library Prep Kit for Illumina (E7645, NEB). DNA libraries were used for DNA libraries.

### 2.9 Statistical analysis

All data are presented as mean ± SEM. Statistics was analyzed by using SPSS 22.0 software (SPSS, Chicago, IL, USA). The unpaired t-test was used for comparisons between two groups, and ANOVA followed by Tukey’s post hoc analysis was used for comparisons between multiple groups or Student’s *t* test (two groups). A probability value *p* < 0.05 was considered to be statistically significant.

## 3 Results

### 3.1 Foxp1 expression levels in patients with bladder cancer

**Figure 1:**
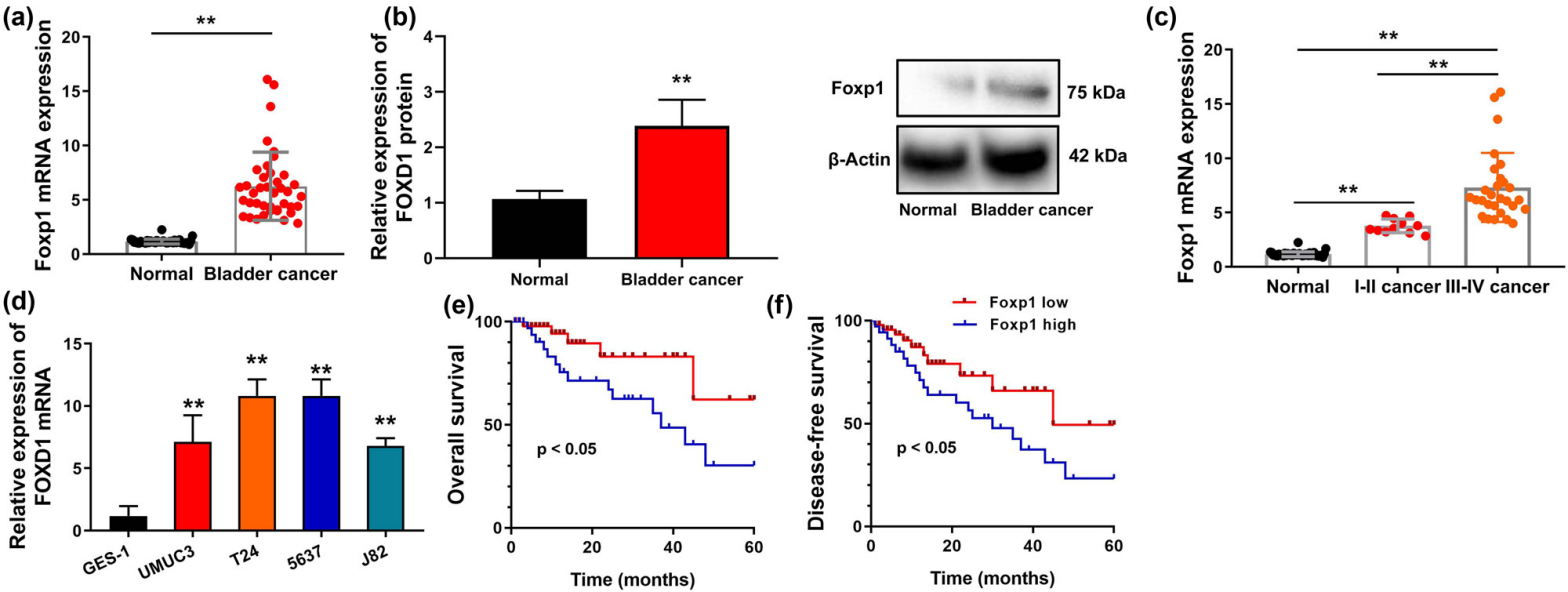
Foxp1 expression levels in patients with bladder cancer. (a–c) Foxp1 mRNA and protein expression in patients with bladder cancer; (d) Foxp1 mRNA in bladder cancer lines; (e and f) overall survival and disease-free survival in patients with bladder cancer. Normal, normal group; bladder cancer, patients with bladder cancer; **p < 0.01 compared with normal group or GES-1.

This work first examines the Foxp1 expression levels in patients with bladder cancer. Foxp1 mRNA and protein expression levels in patients with bladder cancer were increasing, compared with paracancerous tissue (normal) (Figure 1a and b). According to our results, Foxp1 mRNA expression level in paracancerous tissue was lower than that of I–II patients with bladder cancer, and Foxp1 mRNA expression of I–II patients with bladder cancer was lower than that of III–IV patients with bladder cancer (Figure 1c). Foxp1 mRNA expression level in GES-1 cells was lower than those of bladder cancer cell lines (Figure 1d). As a result, OS and DFS of Foxp1 low expression in patients with bladder cancer were higher than those of Foxp1 high expression (Figure 1e and f). Taken together, our data suggest that Foxp1 played a repair factor in bladder cancer.

### 3.2 Foxp1 promoted bladder cancer cell growth *in vitro* model

**Figure 2:**
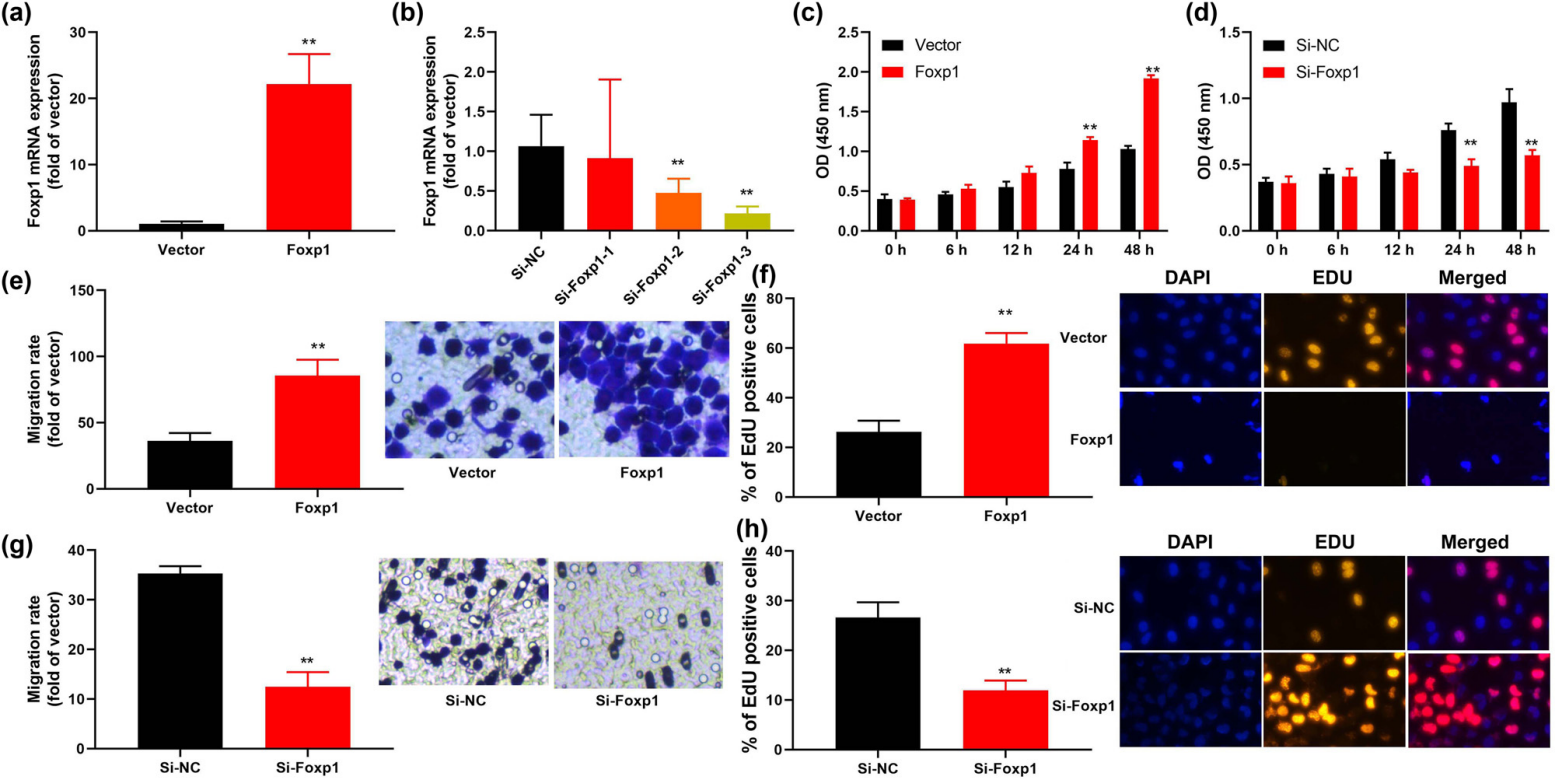
Foxp1 promoted bladder cancer cell growth *in vitro* model. (a and b) Foxp1 mRNA expression, (c and d) cell growth (CCK-8), (e and f) migration rate and EDU assay (d) *in vitro* model of over-expression of Foxp1, (g and h) migration rate and EDU assay (d) *in vitro* model of down-regulation of Foxp1. Vector, negative control group; Foxp1, over-expression of Foxp1 group; Si-nc, si-negative control group; Si-Foxp1, downregulation of Foxp1 group; **p < 0.01 compared with negative control group or si-negative control group.

Thereafter, we examined the function of Foxp1 on cell growth of bladder cancer cell lines. It was found that Foxp1 plasmid increased the expression of Foxp1 mRNA level in bladder cancer cell (Figure 2a). Si-Foxp1 reduced Foxp1 mRNA level in bladder cancer cells (Figure 2b). Over-expression of Foxp1 promoted cell growth of bladder cancer cells, and down-regulation of Foxp1 reduced cell growth of bladder cancer cell (Figure 2c and d). Over-expression of Foxp1 promoted the migration rate and number of EDU cells in bladder cancer cells (Figure 2e and f). Down-regulation of Foxp1 reduced the migration rate and number of EDU cells in bladder cancer cells (Figure 2g and h). Therefore, we focused Foxp1-promoted cell growth and migration rate of bladder cancer.

### 3.3 Foxp1 increased Warburg effect of bladder cancer

**Figure 3:**
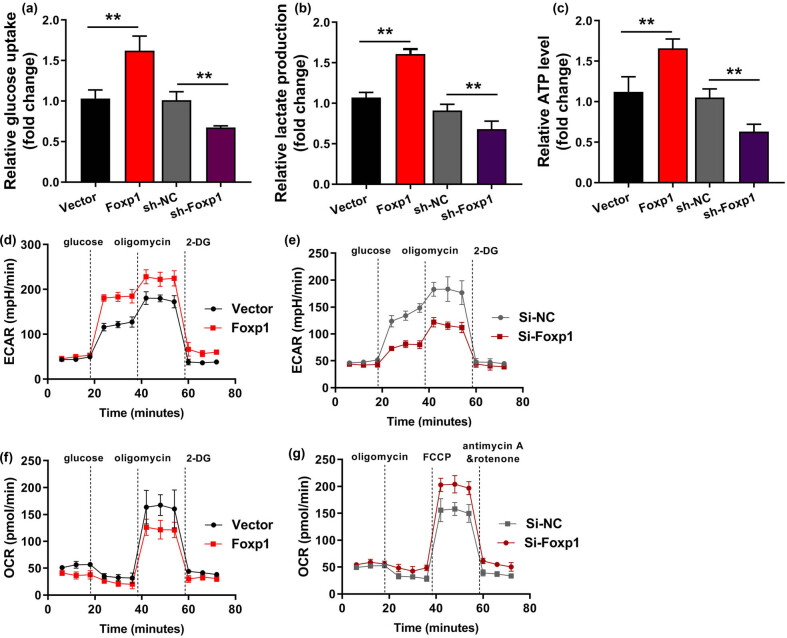
Foxp1 increased the Warburg effect of bladder cancer. Glucose consumption analysis revealed (a) glucose consumption, (b) lactate production analysis revealed lactate production, (c) ATP quantity analysis revealed the ATP quantity, (d and e) ECAR analysis for lactate-induced acidification of the medium surrounding cells, and (f and g) OCR analysis for mitochondrial respiratory capacity was conducted using Seahorse XFp assay. Vector, negative control group; Foxp1, over-expression of Foxp1 group; Si-nc, si-negative control group; Si-Foxp1, downregulation of Foxp1 group; **p < 0.01 compared with negative control group or si-negative control group.

As a next step in investigating the function of Foxp1 on the Warburg effect of bladder cancer. Over-expression of Foxp1 promoted glucose consumption, lactate production, and ATP quantity in bladder cancer cells (Figure 3a–c). Downregulation of Foxp1 reduced glucose consumption, lactate production, and ATP quantity of bladder cancer cells (Figure 3a–c). Furthermore, over-expression of Foxp1 promoted extracellular acidification rate (ECAR) and OCR relative level of bladder cancer cells (Figure 3d and f). Down-regulation of Foxp1 reduced ECAR and OCR-relative levels of bladder cancer cells (Figure 3e and g). These results suggest that Foxp1 could increase the Warburg effect to promote cell growth and migration rate of bladder cancer.

### 3.4 Foxp1 suppressed β-AR expression *in vitro* model

**Figure 4:**
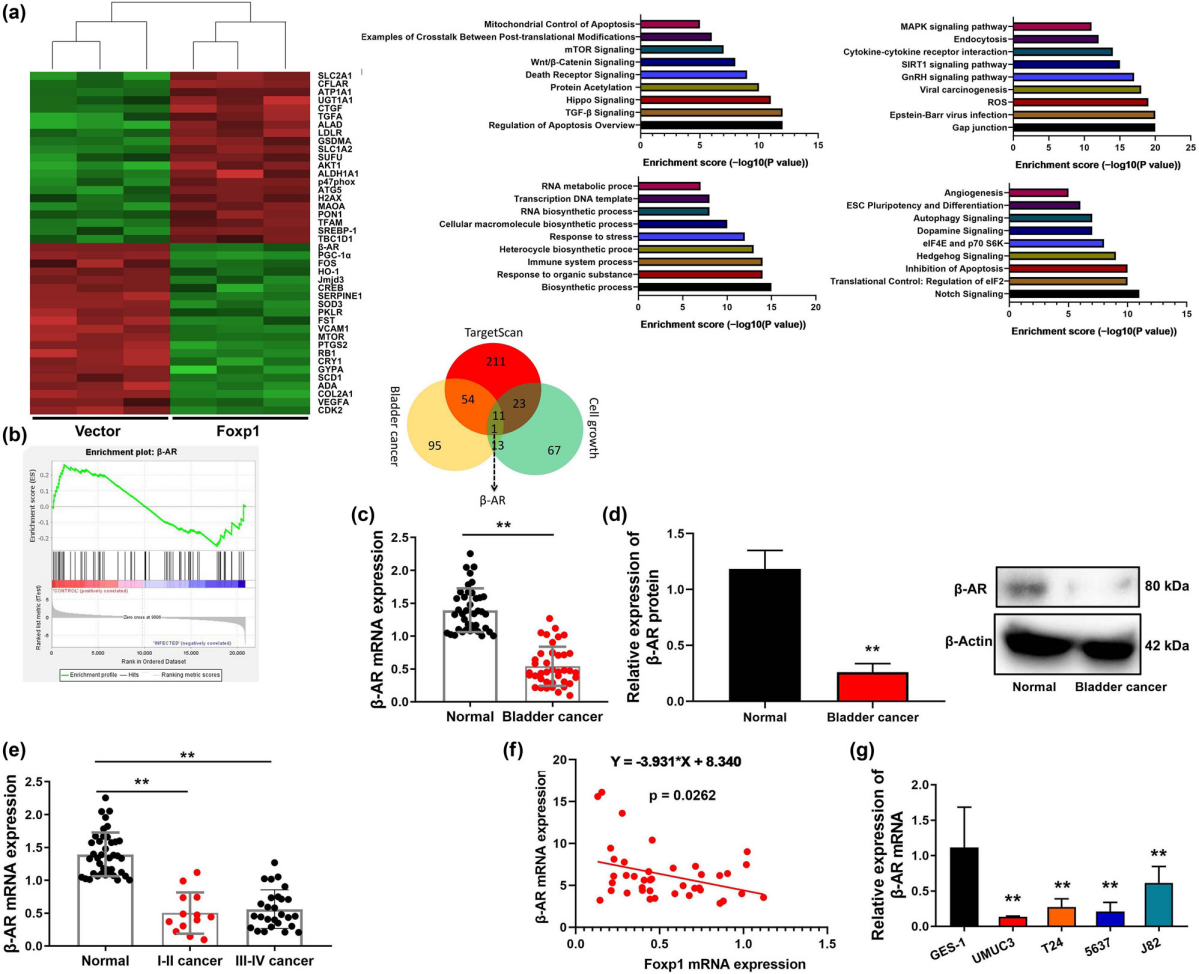
Foxp1 suppressed β-AR expression *in vivo*. Microarray analysis, result diagram, and KEGG terms (a and b), (c–e) β-AR mRNA and protein expression in patients with bladder cancer; (f) correlation between Foxp1 and β-AR in patients with bladder cancer; (g) β-AR mRNA expression in bladder cancer cell lines. Normal, normal group; Bladder cancer, patients with bladder cancer; **p < 0.01 compared with normal group or GES-1.

Subsequently, to observe any potential mechanism of Foxp1 in bladder cancer, we examined gene expression levels by Foxp1 using microarray analysis and β-AR expression may be one important target for Foxp1 on cell growth of bladder cancer (Figure 4a and b). β-AR mRNA and protein expression were reduced in patients with bladder cancer (Figure 4c and d). β-AR mRNA expression level in paracancerous tissue was higher than that of I–II patients with bladder cancer, and β-AR mRNA expression of I–II patients with bladder cancer was higher than that of III-IV patients with bladder cancer (Figure 4e). The mRNA of β-AR was a negative correlation with serum Foxp1 levels in patients with bladder cancer (Figure 4f). β-AR mRNA expression level in GES-1 cells was higher than those of bladder cancer cell lines (Figure 4g).

**Figure 5:**
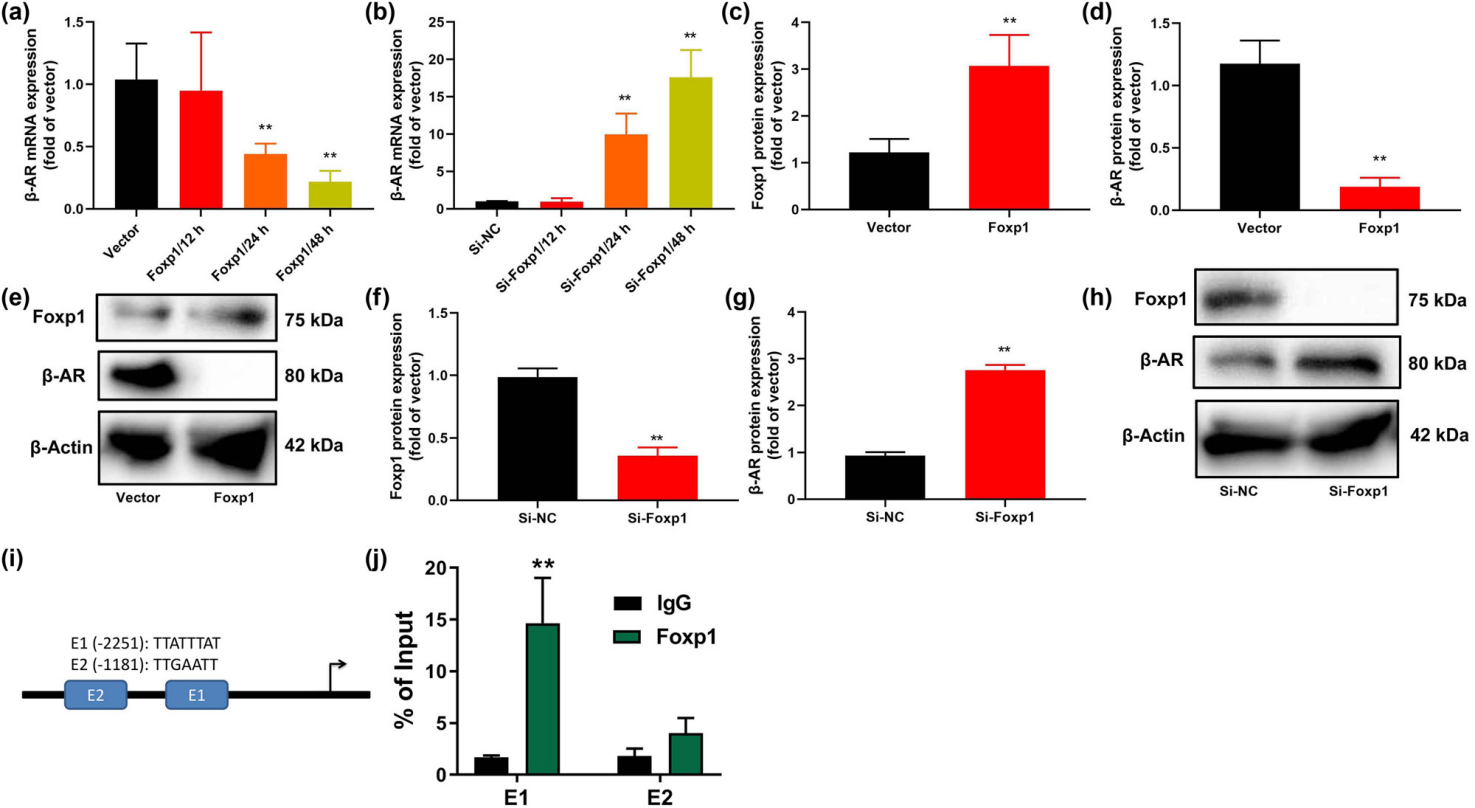
(a and b) Foxp1 suppressed β-AR expression *in vitro* model. β-AR mRNA expression, (c–e) Foxp1 and β-AR protein expression *in vitro* model of over-expression of Foxp1, (f–h) Foxp1 and β-AR protein expression *in vitro* model of down-regulation of Foxp1. Foxp1 binding site at −2,251 bp upstream of β3-AR gene promoter with a schematic drawing (i and j). Vector, negative control group; Foxp1, over-expression of Foxp1 group; Si-nc, si-negative control group; Si-Foxp1, downregulation of Foxp1 group; **p < 0.01 compared with negative control group or si-negative control group.

Next, over-expression of Foxp1 reduced β-AR mRNA expression *in vitro* model (Figure 5a). Down-regulation of Foxp1 induced β-AR mRNA expression *in vitro* model (Figure 5b). Over-expression of Foxp1 induced Foxp1 protein expression and suppressed β-AR protein expression *in vitro* model (Figure 5c–e). Down-regulation of Foxp1 suppressed Foxp1 protein expression and induced β-AR protein expression *in vitro* model (Figure 5f–h). ChIP-seq showed that Foxp1 binding site (E1 and TTATTTAT) was detected at −2,251 bp upstream of the β-AR promoter (Figure 5i and j). In general, data suggest that β-AR is one important targets of Foxp1 in bladder cancer.

### 3.5 β-AR reduced the effects of Foxp1 on cell growth *in vitro* model

**Figure 6:**
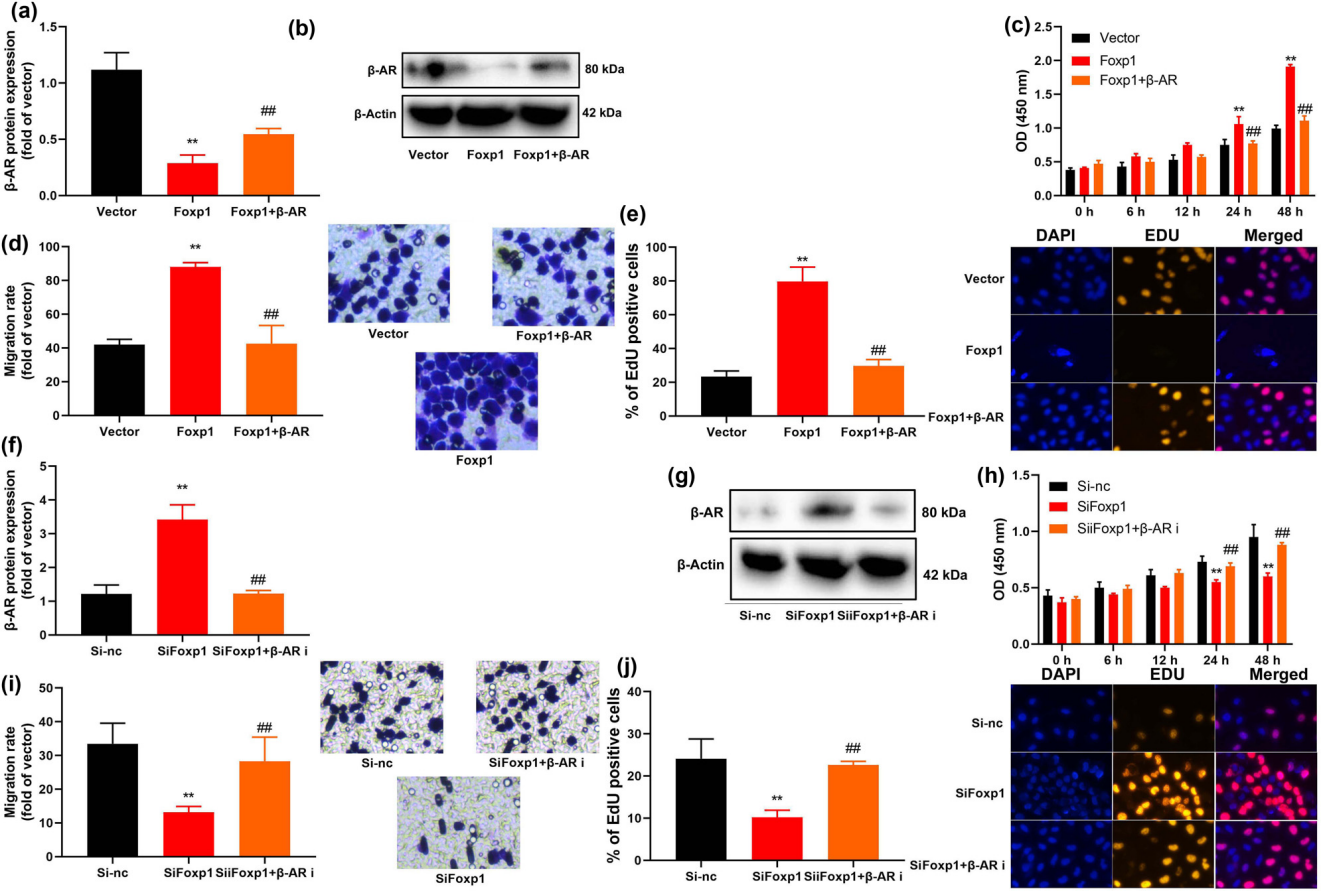
β-AR reduced the effects of Foxp1 on cell growth *in vivo* or vitro model by STAT3 activity. (a and b) β-AR protein expression, (c) cell growth (CCK-8), (d) migration rate, (e) EDU assay *in vitro* model of over-expression of Foxp1 and β-AR; (f and g) β-AR protein expression, (h) cell growth (CCK-8), (i) migration rate, (j) EDU assay *in vitro* model of down-regulation of Foxp1 and β-AR. Vector, negative control group; Foxp1, over-expression of Foxp1 group; β-AR, over-expression of β-AR group; Si-nc, si-negative control group; Si- Foxp1, down-regulation of Foxp1 group; β-AR i, β-AR inhibitor group. **p < 0.01 compared with negative control group or si-negative control group; ^##^ *p* < 0.01 compared with Foxp1 or si-Foxp1 group.

The study determined the role of β-AR controlling the effects of Foxp1 on cell growth and the Warburg effect of bladder cancer. β-AR plasmid increased β-AR protein expression, and reduced cell growth, migration rate and number of EDU cells of bladder cancer cells (Figure 6a–e). β-AR inhibitor Oxprenolol hydrochloride (10 nM) suppressed β-AR protein expression and promoted cell growth, migration rate, and number of EDU cells of bladder cancer cells (Figure 6f–j).

### 3.6 β-AR reduced the effects of Foxp1 on the Warburg effect *in vitro* model by STAT3 activity

**Figure 7:**
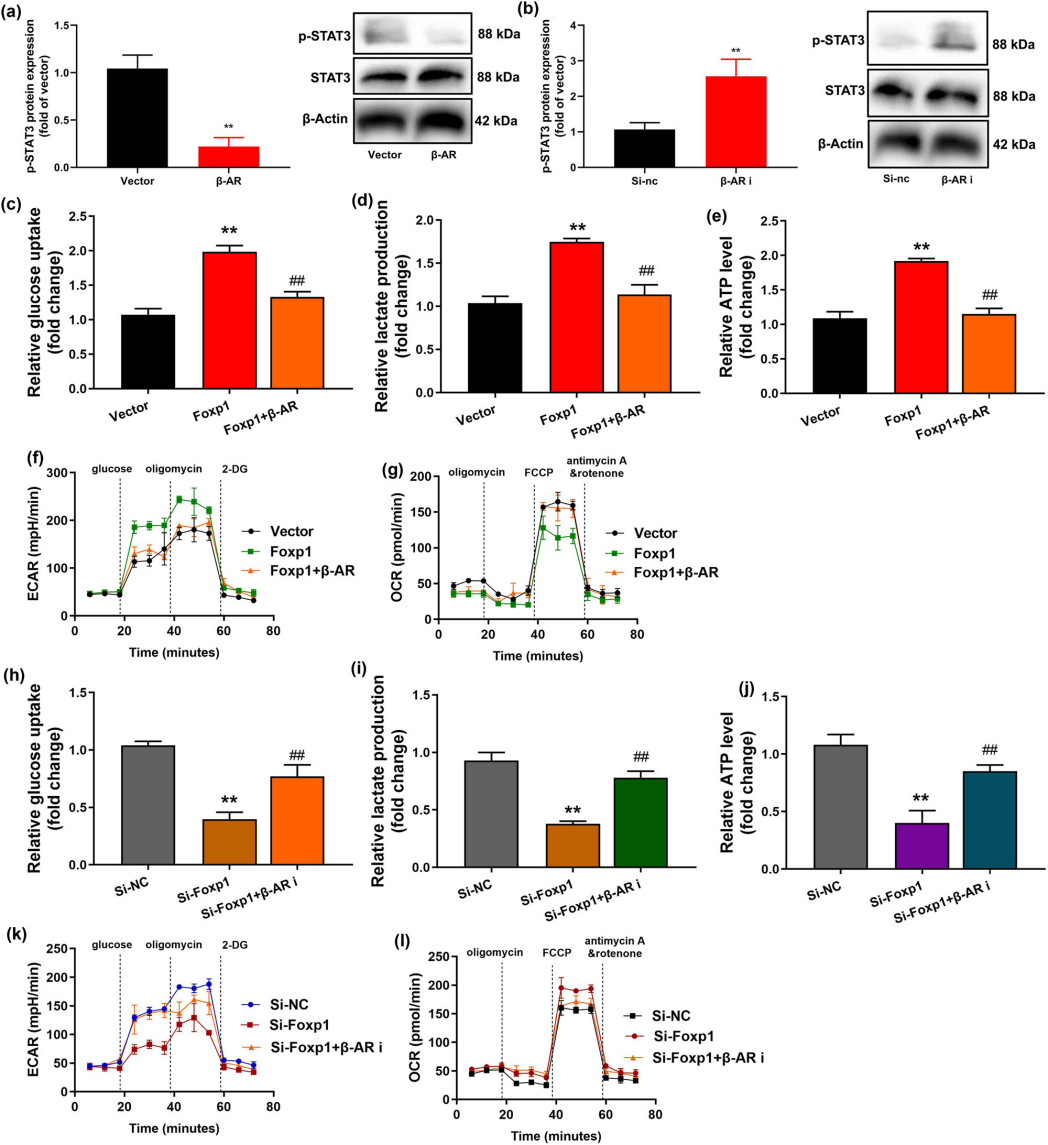
β-AR reduced the effects of Foxp1 on the Warburg effect *in vivo* or vitro model by STAT3 activity. p-STAT3 protein expression (a and b); glucose consumption, lactate production, ATP quantity, ECAR, and OCR relative levels in a bladder cancer cell by over-expression of Foxp1 and β-AR (c–g); glucose consumption, lactate production, ATP quantity, ECAR, and OCR relative levels in a bladder cancer cell by downregulation of Foxp1 and β-AR (h–l). Vector, negative control group; Foxp1, over-expression of Foxp1 group; β-AR, over-expression of β-AR group; Si-nc, si-negative control group; Si- Foxp1, downregulation of Foxp1 group; β-AR i, β-AR inhibitor group. **p < 0.01 compared with a negative control group or si-negative control group, ^##^ *p* < 0.01 compared with Foxp1 or si-Foxp1 group.

Next, this work investigated the mechanism of Foxp1/β-AR on the Warburg effect of bladder cancer. β-AR plasmid suppressed p-STAT3 protein expression in bladder cancer cell (Figure 7a). β-AR inhibitor Oxprenolol hydrochloride (10 nM) induced p-STAT3 protein expression in bladder cancer cells (Figure 7b). β-AR plasmid reversed the effects of Foxp1 on glucose consumption, lactate production, ATP quantity, ECAR, and OCR relative levels in bladder cancer cells (Figure 7c–g). β-AR inhibitor also reversed the effects of si-Foxp1 on glucose consumption, lactate production, ATP quantity, ECAR, and OCR relative levels in bladder cancer cells (Figure 7h–l).

## 4 Discussion

Bladder cancer is one of the common malignant tumors in the urinary system [17]. Non-muscle invasive bladder cancer refers to the bladder tumor confined to the subepithelial connective tissue without muscular invasion [18]. Early diagnosis is currently emphasized internationally [19]. Transurethral bladder tumor resection combined with bladder perfusion therapy in the early stage can prevent the progression and recurrence of non-invasive bladder cancer [19]. In this study, we examined Foxp1 mRNA and protein expression levels in patients with bladder cancer were increased, compared with paracancerous tissue. Wang et al. demonstrated that FOXP1 serves as a prognostic biomarker for gallbladder cancer progression [20]. We suggest that Foxp1 played a repair factor in bladder cancer cell growth.

Warburg effect is known as the anaerobic glycolysis of tumor cells regardless of whether the environment is oxygen-reorganized or not [21]. Warburg effect enables the tumor cells to obtain more glucose and produce energy, thereby promoting metastasis and invasion [22]. In addition, it can lead to an increase of apoptotic resistance of tumor cells [23]. In the glycolysis reaction, the high expression of enzymes such as PDKs and LDH can provide resistance to tumor apoptosis, which can promote the apoptosis of tumor cells separated from the cellular mechanism and prolong the survival period [24]. Surprisingly, we also observed Foxp1 promoted cell growth and increased Warburg effect *in vitro* model of bladder cancer. Fang et al. demonstrated that the novel FOXP1 has a role in renal cell carcinoma progression by the Warburg effect [10]. These results suggest that Foxp1 promoted the Warburg effect of bladder cancer.

β-AR is a multifunctional protein that not only participates in the transduction regulation of the GPCR signaling pathway but also plays the role of signal transduction, scaffold protein, or reactive molecule in other pathways [25]. Because of its important intermediate role in the occurrence, proliferation, and metastasis of tumor cells, it has gradually received attention in the medical field [26]. Our study showed that Foxp1 suppressed β-AR expression *in vitro* model. Foxp1 binding site was detected at −2,251 bp upstream of the β-AR promoter. Liu et al. reveal Foxp1 represses β3-AR transcription [25]. Of note, β-AR it is possible that the effects of Foxp1 may be the promoter factor for bladder cancer progress.

Recent studies have found that STAT3 may promote the Warburg effect in breast cancer and other tumor cells by regulating the expression of glucose transporter 1, pyruvate dehydrogenase, and hexokinase 2 [27,28]. These results of this study suggest that β-AR reduced the effects of Foxp1 on the Warburg effect *in vitro* model by STAT3 activity. Stapel et al. showed that STAT3 expression sensitizes to the toxic effects of β-AR stimulation in peripartum cardiomyopathy [29]. Sun et al. reported that the STAT3 gene was a transcriptional regulator of FOXP1 [30]. Expectedly, Foxp1 promoted STAT3 activity to heighten the Warburg effect by β-AR in a model of bladder cancer.

In conclusion, Foxp1 promoted occurrence and development of bladder cancer through the Warburg effect by the activation of STAT3 activity and repressing β-AR transcription. This experiment submits that further investigation into mechanisms underlying β-AR-mediated STAT3 regulation by Foxp1 will cast new light upon the Warburg effect of therapeutic strategies for bladder cancer. This study provided a new mechanism for understanding the Foxp1-indicated novel target for bladder cancer treatment. Foxp1 is potential target to be used in the treatment of bladder cancer.
